# Test-retest reliability of nerve and muscle morphometric characteristics utilizing ultrasound imaging in individuals with unilateral sciatica and controls

**DOI:** 10.1186/s12998-018-0215-x

**Published:** 2018-11-06

**Authors:** Hadi Sarafraz, Mohammad Reza Hadian, Niloofar Ayoobi Yazdi, Gholamreza Olyaei, Hossein Bagheri, Shohreh Jalaie, Omid Rasouli

**Affiliations:** 1 Department of Physiotherapy, School of Rehabilitation, International Campus, Tehran University of Medical Sciences (IC-TUMS), Tehran, Iran; 2 Department of Neurology, Shahid Mohammadi Hospital, Hormozgan University of Medical Sciences, Bandar Abbas, Iran; 3 Department of Physiotherapy, School of Rehabilitation, Tehran University of Medical Sciences, International Campus (TUMS, IC-TUMS), Brain and Spinal Injury Research Center (BASIR), Tehran, Iran; 4 Radiology, Advanced Diagnostic and Interventional Radiology (ADIR) research center, Tehran University of Medical Sciences, Tehran, Iran; 5 Department of Physiotherapy, School of Rehabilitation, Tehran University of Medical Sciences, (TUMS), Tehran, Iran; 6 Biostatistics, Department of Physiotherapy, School of Rehabilitation, Tehran University of Medical Sciences, (TUMS), Tehran, Iran; 7 Department of Mental Health, Faculty of Medicine and Health Sciences, Norwegian University of Science and Technology (NTNU), Trondheim, Norway

**Keywords:** Reproducibility, Ultrasonography, Skeletal muscle, Low back pain, Sciatic nerve

## Abstract

**Background:**

Ultrasound imaging has been suggested for studying the structure and function of nerves and muscles; however, reliability studies are limited to support the usage. The main aim of this study was to explore the intrarater within-session reliability of evaluating the sciatic nerve and some related muscles morphology by ultrasound imaging.

**Methods:**

Three B-mode images from two scans (transverse and longitudinal) were acquired from the multifidus, biceps femoris, soleus and medial gastrocnemius muscles bilaterally from 15 participants with sciatica and 15 controls in one session, 1-h apart. The data were collected from March to July 2017. Contraction ratio was measured only by longitudinal scan, while the echo intensity was measured using maximum rectangular region of interest in two scans (transverse and longitudinal) for all muscles. Cross-sectional area, direct (tracing) and indirect (ellipsoid formula) methods were used to measure the sciatic nerve. Intraclass correlation coefficient (ICC 3,1), standard error of measurement and minimal detectable change were calculated.

**Results:**

Good to high ICCs (0.80–0.96) were found for muscle contraction ratio in the longitudinal scans in all the muscles in both sciatica and control groups. For echo intensity measurements ICCs ranged from moderate to high, with higher ICCs seen with the maximum region of interest in the transverse scans. The minimal detectable change values ranged between 0.11 and 0.53 cm for contraction ratio.

**Conclusions:**

Ultrasound imaging has high intrarater within-session reliability for assessing the sciatic nerve Cross-sectional area and muscle contraction ratios. Transverse scans with the maximum region of interest result in higher reliability. The sciatic Cross-sectional area is most accurately measured utilizing the direct tracing method rather than the indirect ellipsoid method.

## Background

Low back pain with a clear pathoanatomical diagnosis of lumbar radiculopathy is a common type of specific low back pain (LBP) [1]. Sciatic neuropathy due to disc herniation is the most common peripheral entrapment neuropathy that seen in musculoskeletal setting [2]. Sciatica due to disc herniation compressing a nerve root may lead to disturbance in contractibility of multifidus, biceps femoris, and gastrosoleus muscles innervated by this nerve. To investigate the nerve and muscle morphometric characteristics in sciatica, neuromuscular ultrasound imaging has been suggested for the assessment of both nerve and muscle in patients with entrapment neuropathy [3]. Compared to the magnetic resonance imaging, ultrasound imaging is less expensive, more accessible, feasible and cost-effective [4]. This technique can provide useful information about the muscle function / dysfunction [5]. However, regarding the muscle size, it is important to ensure that the subject is cooperating for either full relaxation or contraction because the muscle dimensions change during contraction / relaxation. Intrarater reliability determines the stability of data recorded by one individual across two or more trials. Having acceptable reliability is essential for any kind of measurement and for making valid decisions [6].

In addition to muscle contraction ratio (i.e. contracted thickness/rest thickness), muscle echo intensity has been recently suggested as a potential marker of muscle-tissue status that can affect the muscle function [7]. Normal muscle displays as a moderately hypoechoic structure in the B-mode ultrasound image because of the low reflection of the ultrasound wave (low echo intensity). Muscles have a speckled look in a transverse scan, due to higher echo intensity of the perimysium around muscle fiber bundles relative to the proper muscle tissue. Longitudinal scans usually have better contrast in echo intensity between the muscle fascicles and the perimysium connective tissue, and this contrast in echo intensity is useful for defining the muscle boundaries and characterization of the muscle architecture [5, 8]. The echo intensity in an ultrasound image can be determined as the average intensity of the pixels inside the target muscle by a scale of gray levels within a given region of interest (ROI) [9]. In the literature, the reliability of echo intensity measures for muscles is controversial and there are still questions regarding the most appropriate method to collect such measures. In addition, the appropriate size of ROI is questionable and some authors such as Caresio et al. (2015) have suggested including as much of a muscle as possible, but avoiding surrounding fascia and bones [7]. Imaging whole section of a muscle may be important since internal fascia and nonhomogeneous distribution of echo intensity might affect the measures. The orientation of muscle bundles might also affect the reliability of echo intensity measures, particularly in longitudinal scans [7].

Sciatica may cause swelling of the sciatic nerve at the posterior thigh level. Increased size and loss of echogenicity of compressed nerves are not well understood. This may partly result from increased vascularity and edema around the nerve. Some studies have investigated the reliability of nerve size and echogenicity of peripheral nerves [10–13]. However, little is known about the reliability of the cross-sectional area (CSA) and echogenicity of the sciatic nerve at the posterior mid-thigh. Moreover, the reliability of measuring nerve enlargement by ultrasound has received little attention in the literature, and there is limited information on the reliability of morphometric characteristics (CSA and echo intensity) of the sciatic nerve, e.g. in patients with sciatica due to disc herniation. One study has investigated the reliability and validity of ultrasound imaging on the sciatic nerve CSA and muscle thickness in healthy subjects with small sample size [14]; however, there is no study conducted to determine the reliability of the sciatic nerve CSA and echo intensity by ultrasound imaging in sciatica patients [15].

Therefore, the main aim of this study was to investigate the intrarater within-day reliability of the nerve and muscle morphometric characteristics in both transverse and longitudinal scans, while utilizing a max rectangular ROI or entire scanned section of the muscle (max ROI) in sciatica patients and controls.

## Methods

### Participants

Fifteen patients, aged 30–50 years with the complaint of LBP with unilateral radiculopathy (sciatica) lasting for a minimum of three consecutive months participated voluntarily in this study (Table 1). Diagnosis was made by a neurosurgeon based on the criteria recommended by Nijs et al. (2015) (LBP with at least one symptom of pain, numbness, or tingling radiating down to the leg and / or foot) confirmed by associated disc bulging or herniation and nerve root compression between the vertebral level at L4-L5 and L5-S1 on MRI [1]. In addition, 15 asymptomatic individuals were recruited from the local population as the control group with no history of LBP during the preceding 6 months, or dysfunction in the low back, thoracic, pelvis, or lower extremities. The control group was matched with the patient group regarding age and gender. Patients completed the Persian version of the Oswestry Disability Index (ODI) [16], Tegner Activity Scale [17] and the Numerical Pain Rating Scale (NPRS) [18]. Tegner Activity Scale is a graduated list of activities of daily living, recreation, and competitive sports; and the score scales from 0 to 10 where 0 represents sick leave or disability pension, and 10 is participation in competitive sports [17]. Patients were excluded if they had tumor / malignancy or bony defects in the lumbar region on MRI, systemic myopathy/neuropathy, previous surgery in the region of assessment, or evidence of central sensitization in the mechanism of pain [1]. All participants signed a consent form and the study was approved by the Human Ethics Committee at Tehran University of Medical Sciences, Tehran, Iran. The data were collected from March to July 2017.

**Table 1:** Demographic data (mean ± SD) of the participants in each group (sciatica and control)

Group	Age (year)	BMI (kg/m2)	Tenger scale^a^	NPRS^b^ back	NPRS Leg	ODI^c^ Range
Sciatica (*n* = 15)	42 ± 14.1	24 ± 2.3	3 ± 0.1	4.8 ± 1.9	5.8 ± 1.6	35.7–40.1 ± 11.9–13.7
Control (*n* = 15)	41 ± 13.9	23.6 ± 2	3 ± 0.1	–	–	–

*BMI* Body mass index; ^a^Tegner Activity Scale, score 0–10 where 0 represents sick leave or disability pension, and 10 is participation in competitive sports. ^b^Numeric Pain Rating Scale, on scale 0–10, a score 1–3 indicates mild pain, 4–6 indicates moderate pain, 7–10 indicates severe pain. ^c^ODI: Oswestry Disability Index, on a percentage scale, scores from 0 to 20% indicate a minimal disability, 21–40% indicate a moderate disability, 41–60% severe disability, 61% to 80% for crippled and 81% to 100%

### Apparatus

A diagnostic ultrasound imaging unit set in B-mode (Affinity 50 Philips-Netherland) with a linear-array probe with 7–12 MHz band frequency was used to record the images. The gain was set at 48% of the range, dynamic range was maintained at 93 dB, and time compensation was kept at the same (neutral) position for all images’ depths. The depth setting was adjusted for each muscle to visualize their superior and inferior margins. Images were recorded as JPEG files and stored on a computer for later processing. ImageJ software (Version 1.48v, National Institutes of Health, Bethesda, MD, USA) was utilized to calculate the muscle thickness and echo intensity as well as the sciatic nerve CSA and echo intensity.

### Data acquisition

**Fig. 1:**
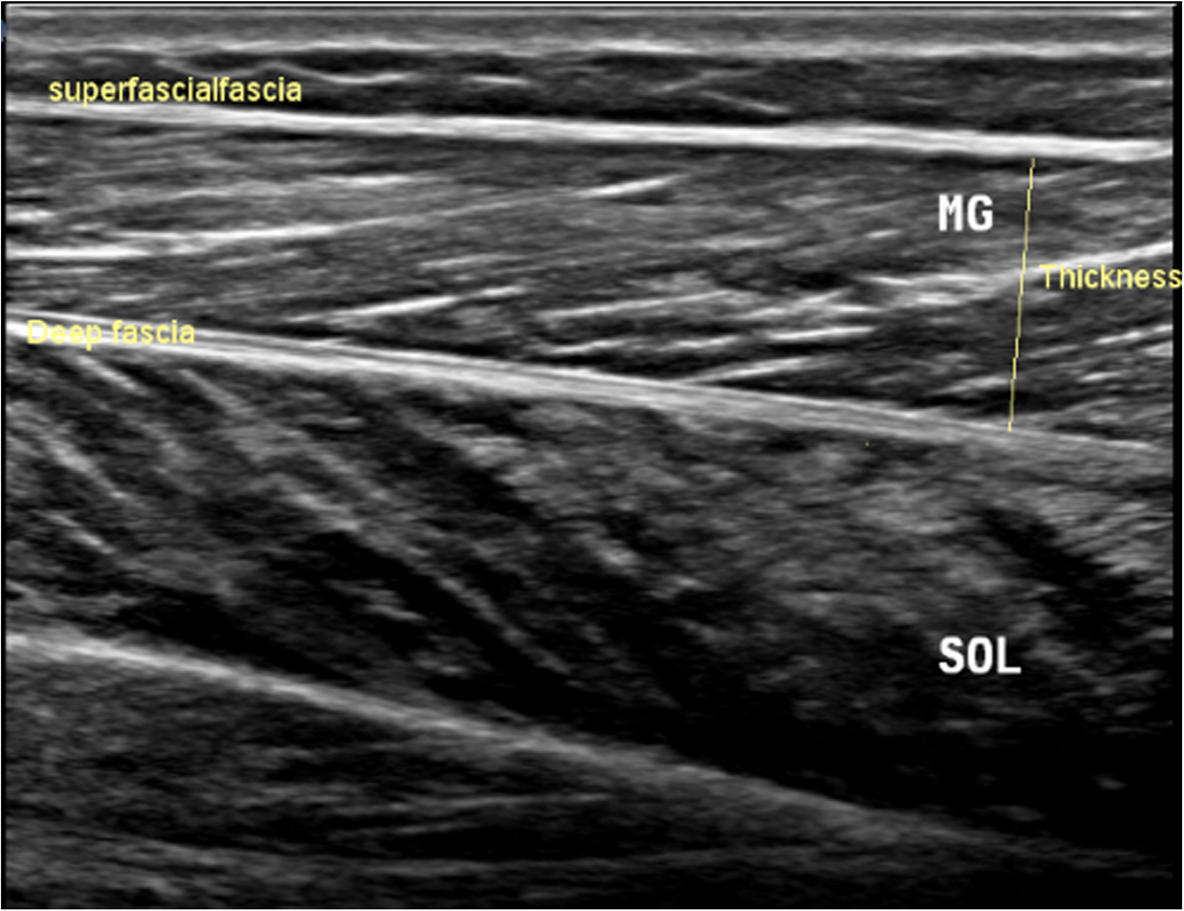
Muscle thickness in the longitudinal scan, the distance between the superficial and deep fascia. In the the longitudinal scan, the US probe is placed parallel to the longitudinal axis of the target structure. MG: medial gastrocnemius muscle, SOL: soleus muscle

**Fig. 2:**
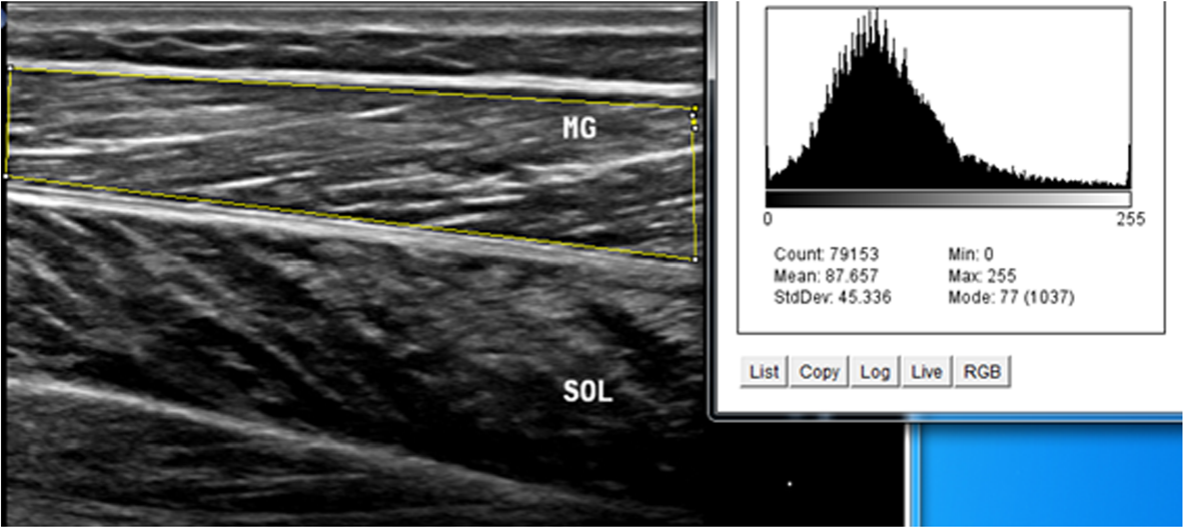
Maximum region of interest (ROI) with histogram echo intensity in the longitudinal scan. A maximum ROI was defined for each image to include as much of the muscle as possible, avoiding bone and surrounding fasciae. Histogram quantifies the greyscale of each pixel in arbitrary units. MG: medial gastrocnemius muscle, SOL: soleus muscle

**Fig. 3:**
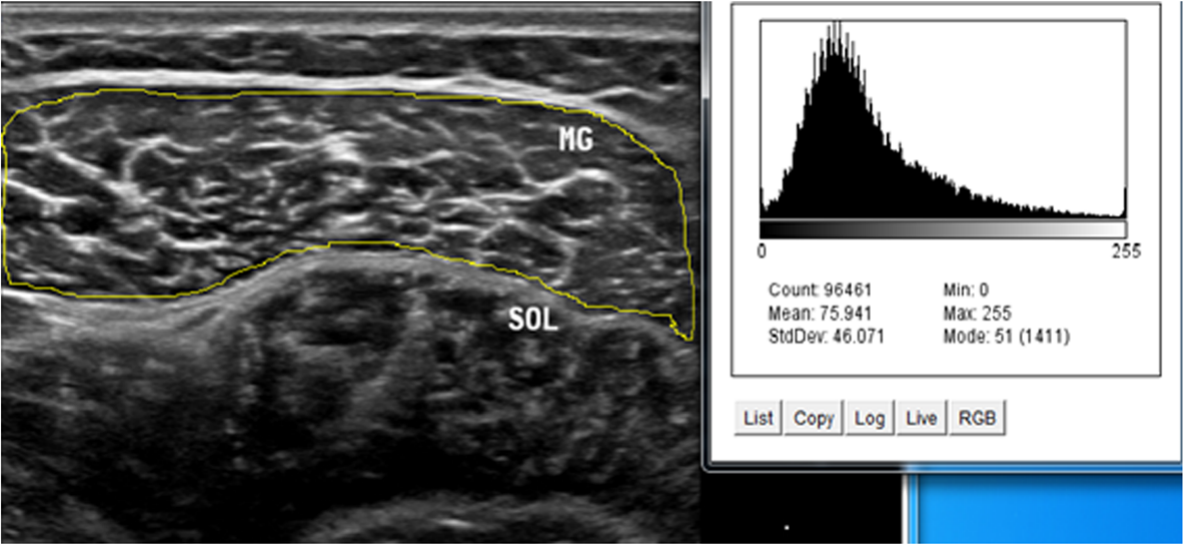
Maximum region of interest with histogram echo intensity in the transverse scan. In the transverse scan, the US probe should be placed perpendicular to the structure of interest. MG: medial gastrocnemius muscle, SOL: soleus muscle

**Fig. 4:**
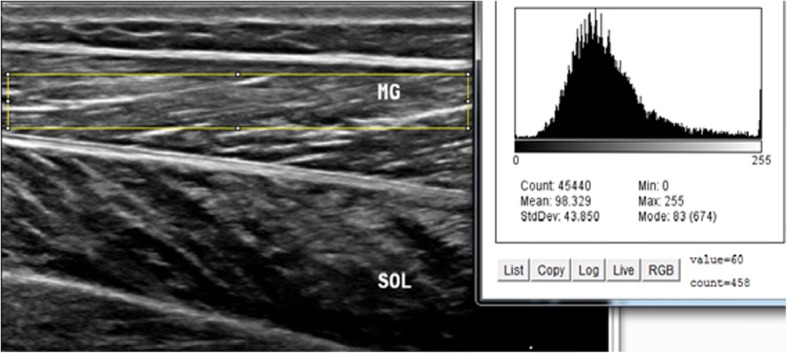
Maximum rectangular region of interest with histogram echo intensity in the longitudinal scan. MG: medial gastrocnemius muscle, SOL: soleus muscle

**Fig. 5:**
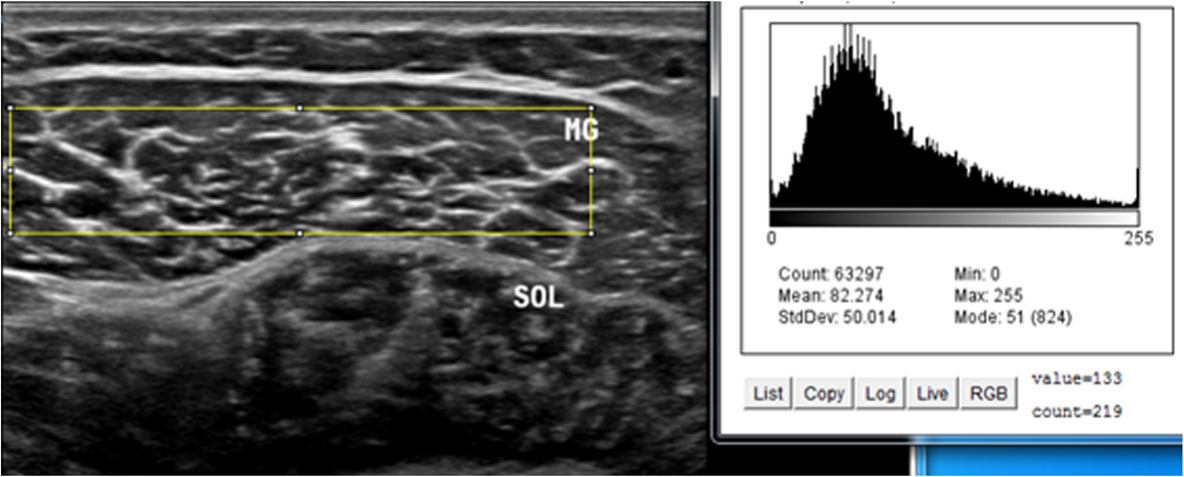
Maximum rectangular region of interest (ROI) with histogram echo intensity in the transverse scan. Rectangular ROI was chosen in each scan to include as much of the muscle as possible without any bone or surrounding fascia. MG: medial gastrocnemius muscle, SOL: soleus muscle

Intrarater within-day reliability of ultrasound measurements of contraction ratio and echo intensity was assessed in all the participants. First, the examiner performed all the measurements, and then, repeated the measurements after 60 min in a random order (condition names picked from a bowl) with the same procedure. To assess the intrarater reliability, three ultrasound images were acquired in the transverse and longitudinal views of four muscles, i.e. multifidus, biceps femoris, medial gastrocnemius, soleus. Between each scan, the probe was moved away from the nerve and muscle and then placed back again over the same area of the nerve and muscle for the next scan. All images were recorded at the end of expiration and they were captured from two sides within one session in the transverse and longitudinal views. Muscle thickness was measured as the largest distance between the superficial and deep fasciae, identified by their hyperechoic appearance in the longitudinal scan (Fig. 1). Two different ROIs were selected from the rest position to measure the echo intensity. First, maximum ROI was drawn for each scan to include as much of the muscle as possible, avoiding bone and surrounding fasciae in two scan views (Figs. 2 and 3). Second, to calculate max rectangular ROI, a rectangular ROI (as large as possible) was positioned over the inner region of muscle image in two scan views (Figs. 4 and 5). Echo intensity was then defined as the mean level of gray within the ROI in 8-bit resolution images (gray levels from 0 to 255, where black = 0 and white = 255) [7].

**Fig. 6:**
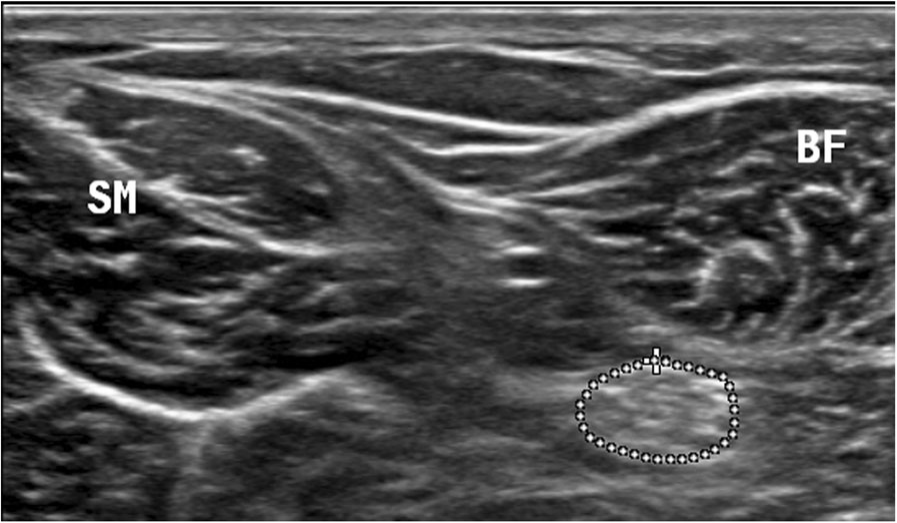
Sciatic nerve with trace cross-sectional area. In the direct method, the inner border of the perineural echogenic rim that surrounds the hypoechoic sciatic nerve was traced, and measured nerve area by tracing along the hyperechoic epineurium, approximating inside of the epineurium. BF: biceps femoris muscle, SM: semimembranosus muscle

**Fig. 7:**
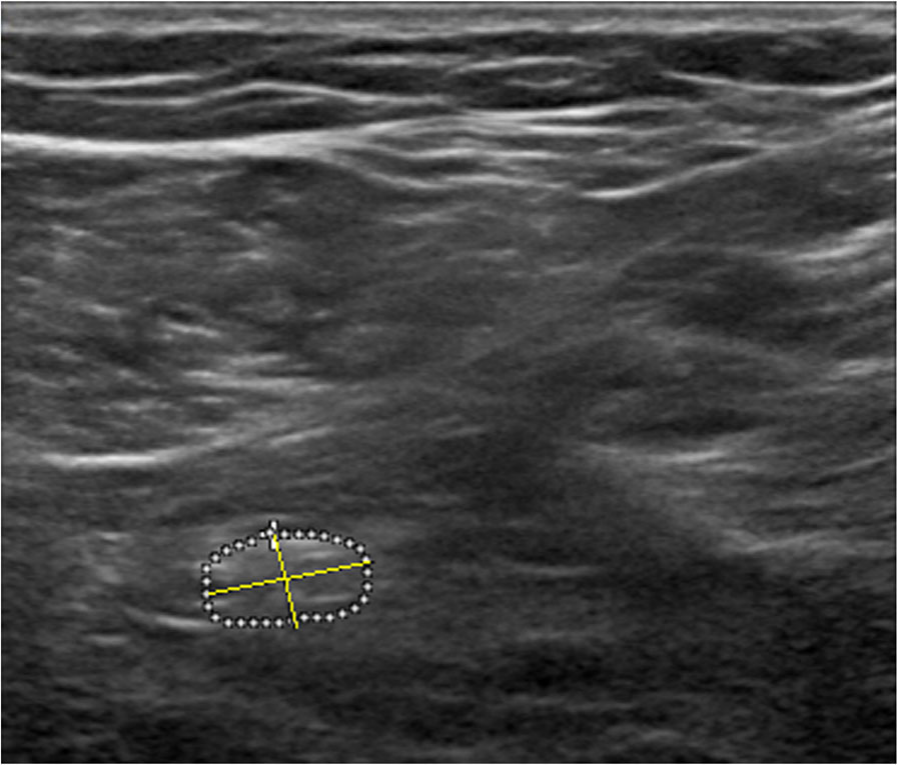
Sciatic nerve with ellipse cross-sectional area. The major and minor diameters and the formula of an ellipsoid to calculate the area (major diameter × minor diameter × 3.14/4)

Direct and indirect methods were used to measure the CSA of the sciatic nerve. In the direct method, the inner border of the perineal echogenic rim that surrounds the hypoechoic sciatic nerve was traced (Fig. 6). The indirect method employed the formula for calculating an ellipsoid area (major diameter × minor diameter × 3.14/4) (Fig. 7) [19]. The major diameter is defined as the longest line between two points of the nerve that passes through the center, whereas the minor diameter is the line through the center of the nerve perpendicular to the major diameter. All images were saved and exported for further analysis and the examiner was blind to the images’ group allocation during processing the images by ImageJ software.

#### Ultrasonography of the sciatic nerve and biceps femoris

Ultrasonography of the sciatic nerve and biceps femoris muscle was performed at the level of lower ¼ on the posterior mid-thigh, along with a line from the ipsilateral iliac crest to the popliteal crease. Longitudinal scans were used to measure muscle thickness, and transverse scans provided a cross-sectional view of the biceps femoris and the sciatic nerve [20]. Participant performed an isometric prone hip extension with a straight knee (off the table to a height of ~ 20 cm) to activate the biceps femoris muscle.

#### Ultrasonography of medial gastrocnemius and soleus

The medial gastrocnemius and soleus muscles were captured at the point of lower 1/3 of the tibial length from the midpoint of the medial malleolus to the popliteal crease [21]. Longitudinal scans were used to record the thickness of the soleus and medial gastrocnemius, while transverse scans provided only a cross-sectional view of the medial gastrosoleus. Participant rose on the toes (5-cm heel lift) while standing to activate the soleus and medial gastrocnemius.

#### Ultrasonography of multifidus muscle

The multifidus muscle was imaged at the L5 vertebral level of the lower back. To record longitudinal scans, the US probe was placed ~ 2 cm lateral to the midline in a way having the spinal facet joints clearly in the image. To record transverse scans, the probe was spanned across the spinous processes to have bilateral cross-sectional views of the multifidus muscles. Participant laid prone and lifted the ipsilateral leg off the table to a height of ~ 20 cm to activate the multifidus muscle [22].

### Statistical analysis

Data were presented as means ± standard deviations (SDs). All statistical analyses were performed with SPSS statistical software version 24 (IBM Corporation, Chicago, IL, USA) software package. Intra-session reliability for the average of three measures in contraction ratio and echo intensity was assessed by the intraclass correlation coefficient (ICC 3, 1; method: alpha, two-way mixed, consistency). The ICCs are classified as follow: < 0.69, poor correlation; 0.70–0.79, fair correlation; 0.80–0.89 good correlation; 0.90–1.00 high correlation [23]. The standard error of measurement (SEM) and minimal detectable change (MDC) were also calculated to make a judgment about the degree that measurements vary for an individual. The SEM values indicate the precision of the measurement and were calculated based on the ICC and the SD of the mean of differences between the two measurements [SEM = SD√1 - ICC]. The MDC represents the smallest change in a score within an individual that can be considered as a real change above measurement error, which was determined using the formula: [MDC = 1.96 × √2 × SEM] [24].

## Results

The demographic data for both patients and controls are summarized in Table 1. Both groups had very similar age and BMI. The sciatica group had moderate to severe pain and disability. Reliability data of muscle contraction ratios are presented in Table 2. Within the patient group, the affected side had a lower contraction ratio with larger SD compared to the unaffected side. The reliability of the muscles was good to high (ICC = 0.82–0.91) in both groups. The SEM and MDC ranged from 0.03–0.08 and 0.07–0.22 in the controls, and 0.04–0.08 and 0.13–0.22 in the sciatica group, respectively.

**Table 2:** Reliability of muscle contraction ratio in the controls (dominant and nondominant sides) and patients with sciatica (affected and unaffected sides)

	Mean	SD	ICC	SEM	MDC
Dominant					
Multifidus	1.24	0.19	0.82	0.08	0.22
Biceps Femoris	1.17	0.14	0.91	0.04	0.11
Medial Gastrocnemius	1.12	0.07	0.90	0.03	0.08
Soleus	1.10	0.06	0.87	0.03	0.07
Nondominant					
Multifidus	1.25	0.17	0.84	0.06	0.18
Biceps Femoris	1.20	0.13	0.90	0.03	0.14
Medial Gastrocnemius	1.12	0.07	0.86	0.03	0.09
Soleus	1.10	0.07	0.88	0.03	0.09
Affected					
Multifidus	0.87	0.40	0.85	0.05	0.16
Biceps Femoris	1.13	0.25	0.89	0.08	0.22
Medial Gastrocnemius	1.01	0.20	0.87	0.07	0.19
Soleus	0.90	0.07	0.90	0.06	0.17
Unaffected					
Multifidus	1.20	0.14	0.89	0.04	0.13
Biceps Femoris	1.27	0.16	0.90	0.04	0.13
Medial Gastrocnemius	1.20	0.21	0.86	0.07	0.19
Soleus	1.25	0.22	0.89	0.06	0.18

*SD* Standard Deviation, *ICC* Intraclass correlation coefficient, *SEM* Standard Error of Measurement, *MDC* Minimal Detectable Change

Table 3 reports the reliability and mean ± SD of two methods (i.e., trace and ellipse) to measure the sciatic nerve CSA as well as echogenicity. In general, the trace method had higher ICC, and lower SEM and MDC compared to the ellipse method. Reliability of echogenicity ranged from 0.71 to 0.74; and 0.75 to 0.82 in the controls and patients, respectively.

**Table 3:** Reliability of sciatic nerve cross-sectional area / echo intensity in the controls (dominant and nondominant sides) and patients with sciatica (affected and unaffected sides)

	Cross-Sectional Area (mm^2^)										Echogenicity (a.u.)				
	Trace method					Ellipse method									
	Mean	SD	ICC	SEM	MDC	Mean	SD	ICC	SEM	MDC	Mean	SD	ICC	SEM	MDC
Dominant	47.76	12.77	0.94	3.10	8.59	47.54	12.59	0.82	5.34	14.76	75.5	26.40	0.74	13.44	37.14
Nondominant	48.01	12.3	0.97	2.14	5.94	47.98	12.51	0.88	4.33	11.96	74.98	21.07	0.71	11.24	31.07
Affected	51.81	10.0	0.91	3.0	8.31	50.97	9.73	0.83	4.01	11.08	70.64	26.48	0.75	13.24	36.59
Unaffected	45.54	9.11	0.90	2.88	7.97	45.11	8.98	0.81	3.91	10.81	71.67	37.28	0.82	15.81	43.70

*ICC* Intraclass Correlation Coefficient, *SEM* Standard Error of Measurement, *MDC* Minimal Detectable Change, *a.u.* arbitrary units

Reliability and mean ± SD of muscle echo intensity measures using max rectangular ROI with two different scans (transverse and longitudinal) are presented in Table 4. The longitudinal scan (0.80–0.89) had slightly higher ICC values compared to the transverse scan (0.75–0.87).

**Table 4:** Reliability of muscle echo-intensity measures for the maximum rectangular region of interest in the controls (dominant and nondominant sides) and patients with sciatica (affected and unaffected sides)

	Transverse scan (a.u.)					Longitudinal scan (a.u.)				
	Mean	SD	ICC	SEM	MDC	Mean	SD	ICC	SEM	MDC
Dominant										
Multifidus	90.53	18.27	0.81	7.95	21.99	91.83	23.20	0.83	9.56	26.42
Biceps Femoris	67.89	21.26	0.76	10.40	28.74	59.08	16.86	0.80	7.54	20.84
Medial Gastrocnemius	79.98	14	0.78	6.57	18.67	84.82	20	0.82	8.48	23.39
Soleus	104.39	47.16	0.79	21.60	59.70	100.74	45.58	0.81	19.83	54.80
Nondominant										
Multifidus	98.13	20.1	0.75	10.05	27.79	89.75	17.31	0.80	7.74	21.39
Biceps Femoris	81.90	23.23	0.77	11.13	30.75	78.74	21.48	0.82	9.11	25.17
Medial Gastrocnemius	79.89	18.67	0.76	9.13	25.23	80.85	14.24	0.83	5.87	16.23
Soleus	104.03	46.29	0.78	16.85	46.57	100.46	48.82	0.82	20.70	57.21
Affected										
Multifidus	99.41	41.53	0.82	17.61	48.69	98.89	41.1	0.84	16.44	45.43
Biceps Femoris	73.71	37.82	0.85	14.64	40.45	80.21	42.72	0.80	19.10	52.79
Medial Gastrocnemius	89.87	45.89	0.83	18.98	52.47	90.55	47.92	0.84	19.17	52.98
Soleus	99.76	31.22	0.87	11.24	31.08	96.63	32.23	0.89	10.67	29.5
Unaffected										
Multifidus	98.74	42.28	0.82	17.93	49.57	99.76	43.87	0.84	17.55	48.50
Biceps Femoris	72.11	39.23	0.80	17.54	48.49	74.10	38.03	0.83	15.67	43.32
Medial Gastrocnemius	80.21	47.97	0.87	17.27	47.72	79.27	48.79	0.86	18.25	50.46
Soleus	95.89	31.52	0.85	12.2	33.51	97.45	35.02	0.84	14.01	38.72

*ICC* Intraclass correlation coefficient, *SEM* Standard Error of Measurement, *MDC* Minimal Detectable Change, arbitrary units (a.u)

Table 5 shows the reliability and mean ± SD of muscle echo intensity measures by maximum ROI with to scans (transverse and longitudinal). There was higher reliability with lower SEM and MDC for muscle echo intensity in the transverse scan relative to the longitudinal scan.

**Table 5:** Reliability of muscle echo-intensity measures for the maximum region of interest in the controls (dominant and nondominant sides) and patients with low back pain (affected and unaffected sides)

	Transverse scan (a.u.)					Longitudinal scan (a.u.)				
	Mean	SD	ICC	SEM	MDC	Mean	SD	ICC	SEM	MDC
Dominant										
Multifidus	64.51	15.1	0.91	4.53	12.51	66.56	23.17	0.80	10.36	28.64
Biceps Femoris	69.28	26.81	0.93	7.08	19.58	59.07	18.56	0.82	7.86	21.73
Medial Gastrocnemius	83.20	19.90	0.96	3.98	10.99	84.45	20.31	0.90	6.42	17.75
Soleus	95.51	46.18	0.94	11.27	31.14	102.50	54.98	0.85	21.28	58.33
Nondominant										
Multifidus	45.29	29.37	0.92	8.29	22.91	66.50	18.37	0.89	6.07	16.80
Biceps Femoris	81.98	20.81	0.94	5.08	14.05	86.32	30.77	0.87	11.08	30.64
Medial Gastrocnemius	79.09	22.43	0.91	6.73	18.57	80.24	16.98	0.92	4.79	13.26
Soleus	106.52	50.06	0.90	15.82	43.72	89.72	49.78	0.88	17.22	47.60
Affected										
Multifidus	108.19	42.37	0.94	10.34	28.58	99.08	36.48	0.82	15.47	42.77
Biceps Femoris	73.32	39.14	0.92	11.04	30.52	78.82	40.37	0.84	16.15	44.63
Medial Gastrocnemius	90.12	45.94	0.93	12.13	33.52	91.36	50.18	0.86	18.77	51.89
Soleus	98.65	30.66	0.91	9.20	25.44	99.18	35.79	0.90	11.31	31.27
Unaffected										
Multifidus	101.90	35.88	0.92	10.12	27.98	106.13	44.59	0.81	19.40	53.61
Biceps Femoris	81.12	38.33	0.92	10.81	29.89	83.48	35.77	0.83	14.74	40.75
Medial Gastrocnemius	81.11	48.82	0.90	15.43	42.66	80.96	49.13	0.87	17.69	48.90
Soleus	99.11	30.26	0.91	9.08	25.10	100.12	33.89	0.89	11.22	31.0

*ICC* Intraclass correlation coefficient, *SEM* Standard Error of Measurement, *MDC* Minimal Detectable Change, *a.u.* arbitrary units

## Discussion

In the current study, ultrasound imaging was used to evaluate the intrarater within-day reliability of nerve and muscle morphological features such as CSA, size, function, and quality of these structures in patients with LBP with unilateral radiculopathy (sciatica) and healthy controls. Acceptable level of reliability is a prerequisite for using ultrasound imaging as a valid measure of muscle activity to make decisions, especially in a clinical setting. In general, the results of this study suggest that there are good to high reliability of ultrasound measurements of the nerve and muscle contraction in healthy controls as well as in the patients with sciatica. These findings support previous studies reporting acceptable reliability for nerve CSA and muscle thickness / echo intensity by ultrasonography [7, 14, 25–27]. However, there are some factors which can affect reliability; for example, examiner’s US experience, participants and the testing situation. Furthermore, several sources of error may affect the ultrasound measurements, such as positioning of the US probe, anatomical landmark detection and precision of marking the fascial bands [28].

### Reliability of contraction ratio

To the best of our knowledge, this is the first study to evaluate test-retest reliability contraction ratio of the lower back and lower limb muscles affected by sciatica. Measuring muscle thickness using rehabilitative ultrasound imaging may be useful as an indicator of muscle function or neuromuscular motor control in the assessment of sciatica due to lumbar disc herniation. The contraction ratio had high reliability and may be used to assess the alterations of muscles morphology and function in patients with sciatica.

Multifidus activation by the abdominal hollowing and contralateral arm rising maneuver has been studied in individuals with and without LBP [29–32]. However, these studies have not used the prone hip extension maneuver to assess multifidus activation. Thus, this method of activation can be employed to contract multifidus in clinical or research settings.

The contraction ratio of measured muscles ranged from 0.87 to 1.27 with large variability. It may be due to the variability of motor control or muscle recruitment patterns in the participants. For example, reduced multifidus muscle contraction ratio on the affected side may result from pain or reflex inhibition due to the nerve root compression. It is also plausible that reduced soleus contraction ratio was a result of individual differences in preferential motor activation and/or co-contraction muscle synergy or antagonist into the muscle chain [33]. Reliability of architectural properties of the medial gastro-soleus had been widely studied previously [34]; however, there is no report regarding the reliability of the muscle contraction ratio of these muscles.

Ruas et al. (2017) also reported utilizing ultrasound imaging as a highly reliable method for the measuring of hamstrings muscle thickness [26]. Individuals may develop a protective motor strategy and adaptation (e.g., co-contraction of agonist-antagonist muscles, or increased activity of synergistic muscles) to pain provocation movement [35]. Due to the high reliability of the muscle contraction ratio, it can be considered as a potential outcome measure when assessing neuromotor function in clinical practice and in research. All the SEMs and the corresponding MDCs were low; thus making the contraction ratio a promising measure for future studies.

### Reliability of sciatic nerve morphological characteristics

The reliability aspect of measuring CSA and echo intensity of the sciatic nerve with ultrasound in patients with LBP with unilateral radiculopathy (sciatica) has received little attention in the literature. This study showed that the sciatic nerve CSA had good to high test-retest reliability, while echo intensity had moderate reliability. The sciatic nerve was not always ellipsoid, therefore the tracing method was a slightly more reliable method than the ellipse method, which is comparable to the previous finding on the measurement of tibial nerve CSA [25]. Therefore, we suggest the direct method to determine the CSA of the sciatic nerve in this population. To our knowledge, little is known about the magnitude of SEM or MDC of the sciatic nerve in these patients. The SEM and MDC of CSA by tracing method were lower than ellipsoid in the controls. A similar trend was observed in the patient group. In line with our findings, two previous studies had reported acceptable reliability when measuring the sciatic nerve CSA [14, 27]. We found moderate reliability for the nerve echo intensity. However, no study has investigated the reliability of the echo intensity of the sciatic nerve in this population. Variability in the MDC measurements of sciatic nerve echo intensity may be due to the echogenic properties of surrounding tissues [13].

### Muscle echo intensity

Muscle echo intensity values might offer important insight into the muscle changes caused by disease / pathological disorders [36], especially because it is more objective and possibly more reliable than a simple visual assessment of ultrasound images [37]. We observed moderate reliability of echo intensity using max rectangular ROI in the transverse scan. This finding is in line with Varanoske et al. (2017), who stated echo intensity may be heterogeneous when examining a portion of individual muscles in the transverse plane [38].

One of our aims was to determine the effect of ROI size on the reliability of ultrasound muscle echo intensity measures. Caresio et al. (2015) reported ICCs of 0.54–0.86 for within-session tibialis anterior and gastrocnemius muscle echo intensity depending on ROI size, with larger ROIs being associated with higher reliability [7]. In our study, using max rectangular ROI displayed lower SEM values for the longitudinal scan but larger SEM values for the transverse scan. Our results also showed that echo intensity reliability was generally lower than the contraction ratio nearly in all the assessed muscles. Lower echo intensity reliability can be due to variability in the US probe placement and potential image-to-image differences in background brightness, which may in return affect the ultrasound absorption and reflection of echo signals [39].

### Limitations

One of the limitations of this study was calculating only intrarater within-day reliability. However, we decided to measure within-day reliability because changes in the hydration level, posture and muscle relaxation between the sessions may affect between-day reliability. Second, this study was performed in the middle-aged population with 1–3 activity level, which limits generalizability to other age groups with higher activity level (e.g., athletes). Another potential limitation was having only one rater, so consistency across multiple raters is unknown.

## Conclusions

This study suggests moderate to high intrarater within-day reliability in muscle contraction ratio and muscle and sciatic nerve CSA and echogenicity in patients with LBP with unilateral radiculopathy and healthy controls. The reliability of echo intensity measurements is sensitive to ROI size; i.e., the maximum ROI in the transverse scan had the highest reliability. These findings can contribute to support the use of ultrasound for reliable evaluation of neuromuscular morphology and particularly to assess the small changes in both nerve and muscle structures in LBP with unilateral radiculopathy.

## Abbreviations

CSA: Cross-sectional area
ICC: Intraclass correlation coefficient
LBP: Low back pain
MDC: Minimal detectable change
ROI: Region of interest
SD: Standard deviations
SEM: Standard error of measurement
